# Handwashing Message Type Predicts Behavioral Intentions in the United States at the Beginning of the Global COVID-19 Pandemic

**DOI:** 10.3389/fpubh.2021.583491

**Published:** 2021-05-05

**Authors:** John Matkovic, Kelly S. Clemens, Kate Faasse, Andrew L. Geers

**Affiliations:** ^1^School of Population Health, University of Toledo, Toledo, OH, United States; ^2^Department of Psychology, University of Toledo, Toledo, OH, United States; ^3^School of Psychology, University of New South Wales, Kensington, NSW, Australia

**Keywords:** COVID-19, social marketing, handwashing, emotion, exchange theory, intentions

## Abstract

Handwashing has been widely recommended to reduce the spread of COVID-19. Despite this, handwashing behavior remains low in the general public. Social marketing has been employed as a successful health promotion strategy for changing many health behaviors in the past. The present study examines if message framing influences the effectiveness of a handwashing health promotion messages at the early stages of the COVID-19 pandemic. In a between-subjects cross-sectional experiment, participants (*N* = 344) in the United States were randomly assigned to view one of four handwashing messages or a control message before completing self-report measures of attitudes, emotions, readiness to change, and behavioral intentions around handwashing. Simple handwashing messages were presented with different framings, including a simple exchange message, a gain message, a social norm appeal, and a guilt appeal. Results revealed that message type influenced handwashing behavioral intentions and emotions. *Post-hoc* comparisons revealed that the simple exchange message produced significantly higher intentions than other messages and that only the simple exchange message significantly differed from the control message on emotions regarding handwashing. Mediational analyses showed handwashing emotions fully mediated the relationship between messaging and handwashing intentions. This mediation effect was moderated by age, such that it occurred for the younger and middle age participants, but not older participants. These results suggest that even simple, brief, and easily conveyable messages can positively impact behavioral intentions around handwashing during the early stages of a health crisis. Consistent with recent research comparing affective and cognitive pathways for health behavior, the mediational analysis suggests that effect of the simple exchange message on intentions was due to increased positive emotions around handwashing.

## Introduction

The Novel Coronavirus 2019 (COVID-19) has undeniably affected life in the United States and around the world. The United States has suffered from increased unemployment, disruptions to educational and leisure activities, and economic struggles. The United States has disproportionately high rates of morbidity and mortality due to COVID-19; despite having only 4% of the world's population, as of July 2020 the United States is responsible for 25% of the world's COVID-19 cases (1). This prevalence of COVID-19 and the concerted efforts to educate the public on its prevention has affected public awareness of preventive health behaviors, including handwashing (2).

Handwashing is a frequently recommended way of preventing disease and reducing the spread of illness and has been touted as a way to prevent spreading COVID-19. The Centers for Disease Control and Prevention (3) recommends individuals wash their hands for at least 20 s, ensuring all skin on the hands is washed. The CDC guidelines also include recommendations for washing hands after visiting the bathroom, before and after eating, and after touching one's face (3, 4). Proper handwashing has long been shown to reduce bacterial load on hands and to reduce the risks of contracting gastrointestinal and respiratory illnesses (5–7). Additionally, and vital to the context of COVID-19, handwashing has previously been associated with a reduced risk of contracting influenza (8). While data related to handwashing and COVID-19 is still emerging, at least one 2020 study shows a correlation between *interest* in handwashing, as measured by Google searches, and reduced COVID-19 spread (9).

Despite the numerous protective benefits associated with handwashing, many still do not perform proper handwashing behaviors. Prior to COVID-19, only two-thirds of people self-reported washing their hands after using public restrooms or after coughing or sneezing, and approximately one third of people wash their hands after shaking hands (10). Healthcare settings are vulnerable to low handwashing compliance as well. The CDC estimates that healthcare workers wash their hands <50% of the time in daily situations where handwashing is recommended (3).

These data suggest that more work must be done to change handwashing behaviors in the United States. However, widespread health behavior change is challenging to achieve. Due to the difficulty inherent in changing behaviors, health promotion interventions often utilize *social marketing* to encourage behavior change. The social marketing approach uses traditional marketing strategies of exchange—where an individual pays some cost to receive some benefit— to promote behavior change (11). *Exchange theory* sits at the heart of social marketing. As in traditional marketing, exchange theory explains that for a behavior to occur, the intended audience must find the desired behavior to be equal to, or greater in value than, the cost to perform the behavior (12). This exchange applies to social marketing as well; the intended population must find the benefits to themselves (or to society) are worth the cost of performing a given behavior (12). To achieve successful exchange, social marketing researchers have developed a variety of approaches for framing health message. Social marketing utilizing exchange theory has been a tool in health promotion and has been successful in improving health behaviors and its use has increased self-reported handwashing in the past (13–15).

In this study four specific message types were compared to a no-message control: gain framing, social norm, guilt appeal, and simple exchange. First, *gain framin*g is a well-known approach to health promotion campaigns. Gain frames highlight how an individual will benefit, or what they will gain from performing a behavior, as opposed to what negatives they will avoid (16, 17). *Social norms approaches* share behavioral information about a given population in an effort to encourage behavior change. For example, social norms have strong influences on behaviors like cannabis use and healthy eating (18, 19). There is evidence of the efficacy of social norms messaging for encouraging positive health behaviors (20), and thus may present a powerful type of message for increasing handwashing (21). *Guilt appeals* are messages that highlight the inherent desire to fix previously immoral, inappropriate, or unhealthy behaviors (22). Antonetti et al. (23) explain that guilt appeals can successfully help change relational or social behaviors. Finally, simple *exchange messages* make clear both the cost of the behavior, and the benefit.

Although frequently effective, social marketing is not a one-size fits all tool. Differences in barriers, awareness, skills, and social and built environments can affect how individuals receive various social marketing messages, and their readiness for behavior change. For this reason, successful marketing is achieved by aligning social marketing messages with the target population in a given context (12). The primary goal of the present research is to compare the effectiveness of four different social marketing message frames for improving responsiveness to a handwashing message in the United States as the beginning of the COVD-19 pandemic.

While social marketing messaging is known to impact behavior change, the mechanism for why this occurs is also still unclear. Many health behavior theories consider behavior change to occur due to cognitive mechanisms, such as attitudes [e.g., (24, 25)]. Recent research suggests that affective or emotional responses may also be important independent mechanisms of change in intentions and behavior [for a review, see (26)]. The present study hypothesizes that the cognitive variable of attitudes and the affective variable of emotions will be unique and independent mediators of the relationship between message framing and behavioral intentions and readiness to change.

The present study also utilizes the Transtheoretical Model (TTM) to examine handwashing behavioral intention. The TTM is a model that describes behavior as traveling through a series of stages—precontemplation, contemplation, preparation, action, maintenance, and termination (27–29). While originally developed after examining the behaviors of smokers who had successfully stopped smoking (27), the transtheoretical model had since been applied to numerous other areas of health behavior change (30). Understanding the stage in which an individual resides is important when creating behavior change messages; thus, the present study explores if readiness to change varied based on different message types.

Finally, due to extensive media coverage of age differences in the effects of COVID-19 age was considered as a possible moderating variable. Discussions around the greater danger of COVID-19 to older Americans may have resulted in the false belief that younger people do not need to increase behaviors like handwashing to the same extent of older people. Previous studies on beliefs about preventative pandemic behaviors demonstrate this; college-aged participants believed that children and the elderly were most vulnerable to disease, and that teens and young adults were less at risk for contracting a pandemic influenza (31). In the current pandemic, there is evidence of younger Americans disregarding health recommendations in order to attend social events (32–34), resulting in new outbreaks of COVID-19 cases. Given this, it is important to learn the impact of age on intentions to take preventative actions, like handwashing.

The present study compares four different social marketing messages for increasing handwashing attitudes, readiness to change, emotions, and behavioral intentions in order to better understand the most effective way to facilitate adherence to handwashing guidelines during the COVID-19 pandemic. Based on these social marketing principles, new messages were created for this study. Further, the study explores whether attitudes or emotions may serve as mediators of the effect of the messages on behavioral intentions and readiness to change. The study also examines the possibility that age moderates the effectiveness of the handwashing messages. Some variables included in this study, such as the demographic items, handwashing attitudes, emotions, intentions, and readiness to change, have been presented in a paper by Clemens et al. (35). The present experimental study focuses on a handwashing message manipulation and its effect on handwashing-related variables such as intentions. While there is overlap in data, separate, a priori hypotheses and analyses were used in each paper.

## Methods and Materials

The present between-subjects cross-sectional design experiment utilized Qualtrics to randomly display a simple message manipulation in order to determine the effect of message type on measures capturing handwashing related attitudes, readiness to change, emotions, and behavioral intentions. All methods and measures were approved by the University of (name redacted for blind review) Social, Behavioral, and Educational institutional review board (IRB protocol number: 300597) and were conducted in compliance with the guidelines of the American Psychological Association.

### Participants

Participants (*N* = 344) were recruited via Prolific (an online participant recruitment system) until available budget was exhausted. All participants received monetary compensation for their time. Of these participants, 54.1% identified as women, 43.9% identified as men, and 1.5% identified as another gender or preferred not to disclose their gender. Participants ranged in age from 18 to 74 (*M* = 32.69, *SD* = 11.60). Participants represented 44 of the 50 United States and were 68% White, 16% Asian/Asian-American, 5% Black, 5% Latinx, and 6% two or more races. For participant income level, 23.7% reported household income lower than $30,000, 37.5% reported between $30,000 and 69.999, 18.9% reported between $70,000 and 99,999, and 19.9% reported income of $100,000 or more. Participants were recruited via Prolific to represent, as closely as possible, the general population of the United States.

### Measures and Materials

#### Demographics

A demographic questionnaire included questions regarding age, race and ethnicity, geographic location, and income level of participants. This questionnaire was also used to capture information about COVID-19 in the participant's community, however these data were not included in the present analyses. Demographic items were shown at the end of the study to avoid the potential of influence on primary dependent measures. See Supplementary Materials for copies of the measures.

#### Handwashing Messages

**Figure 1:**
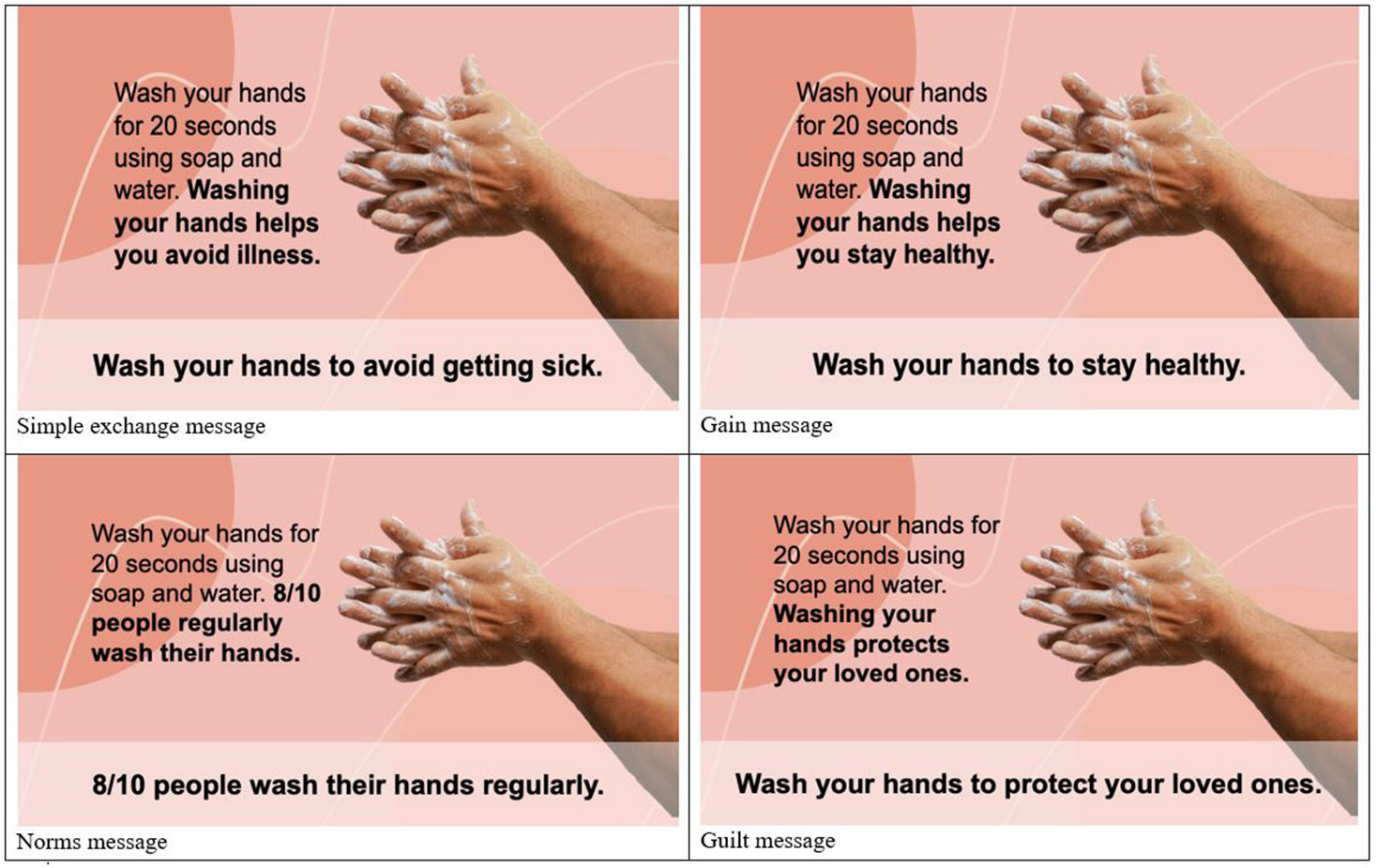
Handwashing messages shown to participants. The top left shows the simple exchange message, the top right message shows the gain message, the bottom left message shows the norm message, and the bottom right message shows the guilt message. Similar images were used.

Participants were randomly assigned to view one of five messages at the beginning of the study. Messages included either one of four handwashing message approaches described earlier: gain framing, social norm, guilt appeal, and exchange or a fifth control message. All five messages were shown with identical graphics. The control message (*n* = 73) simply read, “Press the arrow to continue.” The different messages can be viewed in Figure 1. The four handwashing messages utilized different framings often used in exchange theory social marketing: a gain frame (*n* = 66), a social norms approach (*n* = 68), a simple exchange message (*n* = 65), and a guilt appeal (*n* = 70).

#### Handwashing Attitudes

Participants' attitudes toward handwashing were evaluated using a two-item measure. Items such as “handwashing is important” were scored on a seven-point Likert scale ranging from (1) *strongly agree* to (7) *strongly disagree*. Items asked participants to rate the importance and effectiveness of handwashing in preventing COVID-19. Items used in this measure demonstrated acceptable internal consistency with a Spearman-Brown reliability coefficient of 0.78.

#### Handwashing Emotions

Handwashing emotions were measured using five items assessing discrete emotions related to handwashing. Scale items included emotions of both positive and negative valences, such as “I am proud of washing my hands” and “I would feel guilty if I did not wash my hands.” Items were scored using a seven-point Likert scale ranging from (1) *strongly disagree* to (7) *strongly agree*. This scale demonstrated strong internal consistency (α = 0.83).

#### Handwashing Intentions

Handwashing intentions were measured using six items targeting intention to wash one's hands in scenarios recommended by the CDC and according to the guidelines they provided, such as “after blowing your nose, coughing or sneezing” and “for at least 20 seconds each time.” Items were reported on a five-point Likert scale ranging from (1) *never* to (5) *always*. The scale demonstrated high internal consistency (α = 0.80).

#### Handwashing Readiness to Change

Participants' stage of change was determined using a single item modeled on the work of (36). Participants were asked to select the option which best reflected their intention to wash their hands according to CDC guidelines (e.g., for 20 s multiple times daily). Response options included “I do not intend to do this,” “I have thought about doing this, but do not yet plan to,” “I intend to do this, but have not done it yet,” “I am actively doing this,” and “This is something that I have done for a long time, and intend to continue doing to prevent disease.”

#### Data Analysis

Statistical analyses conducted included correlations between all focal variables and standard one-way ANOVA tests to examine the influence of each message on the variables of handwashing intention, emotions, attitudes, and readiness to change. Welch ANOVA and Games-Howell *post-hoc* tests were conducted when tests violated the assumption of homogeneity of variance. The PROCESS macro for SPSS (37) was used to test for mediation by emotions and attitudes and for the possible moderation by participant age. Data has been made available on the Open Science Framework.

## Results

Means, standard deviations, and correlations between the variables are shown in Table 1. Handwashing intentions, readiness for change, attitudes, and emotions were all positively correlated. Further, handwashing intentions and readiness to change increased with participant age.

**Table 1:** Means, standard deviations, and correlations between measures.

	***M***	***SD***	**1**.	**2**.	**3**.	**4**.
Handwashing intentions	4.45	0.59	–			
Handwashing emotions	5.47	1.07	0.48^***^	–		
Handwashing attitudes	6.67	0.50	0.41^**^	0.39^***^	–	
Handwashing readiness to change	4.25	0.77	0.47^***^	0.33^***^	0.22^***^	–
Age	32.69	11.60	0.18^**^	0.08	0.10	0.11^*^

* *p < 0.05*,

** *p < 0.01*,

*** *p < 0.001*.

Preliminary analyses showed that standard ANOVA tests violated the assumption of homogeneity of variance. As such, Welch ANOVA, which does not assume equal variance, was employed. Significant omnibus tests were followed up with Games–Howell *post-hoc* comparisons for unequal variances. A series of one-way ANOVAs revealed that message type influenced handwashing behavioral intentions, Welch's *F*_(4, 166.59)_ = 4.38, *p* = 0.002, and emotions, Welch's *F*_(4, 168.13)_ = 3.11, *p* = 0.017, but had no statistical impact on handwashing attitudes or readiness to change. As displayed in Table 2, *post-hoc* comparisons revealed that the social exchange message produced significantly higher intentions than the control message, gain message, or guilt appeal, and was the only message type to change intention compared to the control message. Similarly, *post-hoc* comparisons revealed that only the exchange message significantly differed from the control message on emotions regarding handwashing (e.g., “I am proud of washing my hands”).

**Table 2:** Means and standard deviations on intentions and emotion measures and significant condition differences on these measures from the simple exchange message condition.

**Construct**	**Message**	***M***	***SD***	***p***
Handwashing intentions	Simple exchange message	4.66	0.39	–
	Norm appeal	4.46	0.53	0.083
	Gain message	4.38	0.65	0.028^*^
	Guilt message	4.38	0.63	0.019^*^
	Control message	4.40	0.68	0.048^*^
Handwashing emotions	Simple exchange message	5.81	0.83	–
	Norm appeal	5.36	1.08	0.060
	Gain message	5.51	1.08	0.376
	Guilt message	5.44	1.06	0.170
	Control message	5.28	1.20	0.026^*^

*p-values indicate differences from the simple exchange message*.

* *p < 0.05*.

### Mediational Analyses

**Figure 2:**
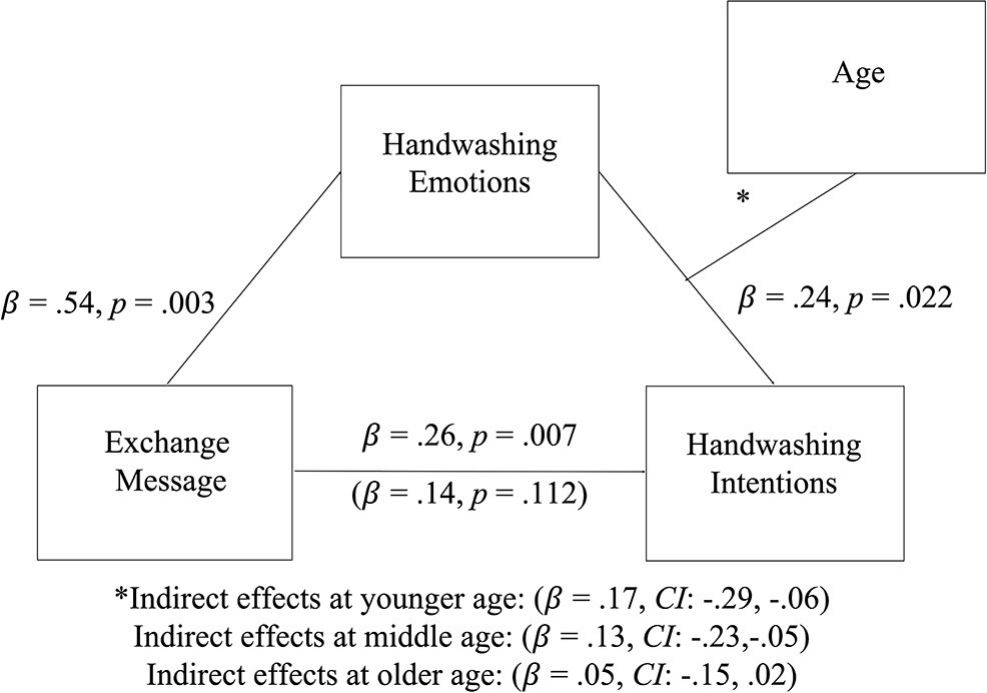
The mediational model.

Mediational analyses (37) were then conducted to determine if the exchange message predicted intentions as a result of handwashing attitudes and emotions. Handwashing emotions (and not handwashing attitudes) completely mediated the relationship between messaging (coded as those who received the simple exchange message and those who received a control message) and handwashing intentions (see Figure 2).

Critically, this mediation effect was moderated by age, such that it occurred for the younger [95% CI (−0.29 to −0.06]), and middle age participants [95% CI (−0.23 to −0.05)], but not older participants [95% CI (−0.15 to 0.02)]. That is, the conditional process analysis, indicated that the exchange message escalated feelings about handwashing which, in turn, increased handwashing intentions for younger and middle-aged adults. This model resulted in a significant index of moderated-mediation, 95% CI (0.01 to 0.13) (38). Exploratory analyses with other demographic variables are presented in the Supplementary Materials.

## Discussion

This study demonstrates that the type of exchange theory-based social marketing message influences handwashing emotions and behavioral intentions, but does not significantly impact handwashing attitudes or readiness to change. Participants who were shown a simple exchange message, a message where a clear benefit and avoided consequence were given, were found to have more positive handwashing emotions and to have higher intentions to wash their hands than those shown other messages or a control message.

This study also suggests different message types housed under the exchange theory can produce different results. The first objective of this study was to compare the ability of four different approaches (vs. a control condition) of presenting a simple handwashing message to change intentions and readiness to change. This aligns with findings from previous handwashing message studies, such as that by (39), which find differential effects of brief handwashing message types, and adds evidence for this in the context of a global health emergency. Simple exchange message were found to be the most effective in increasing handwashing emotions and behavioral intentions. The other three messages compared did not significantly differ from one another on any of the dependent measures. While it is unclear why these messages were the most effective, it may be that the media's focus on the dangers of COVID-19 resulted in the simple exchange message seeming most valuable. The simple exchange message highlights the extra time spent washing hands (the cost) to avoid illness (the benefit). This highlights the avoidance of illness as the benefit, as opposed to maintaining health, being like others, or protecting loved ones, and may connect individuals more to the actual danger presented by COVID-19, thus increasing handwashing related intentions and emotions.

There are other possible reasons why the simple exchange message was found most influential. It is possible the spread of COVID-19 and the subsequent stay-at-home orders in the US created a natural experiment in which the exchange message was most effective. People were overwhelmingly at home, and the inconvenience and time spent washing hands may have been less of a barrier. Further, the social norms message might have resonated less because people were more separated from their social groups. The guilt appeal may have not been as effective because negative emotional appeals like guilt appeals can often have the opposite effect. Individuals instead ignore the message if it comes across as too powerful or too weak (23, 40). Negative emotional appeals like the guilt appeal may still work, but they may require additional work to find the middle ground that is appealing to audiences. This line of research would benefit from future studies exploring both why, when, and for whom these differences occur. Future studies could also more directly examine when a guilt appeal may be effective. Subsequent studies could also benefit from manipulation checks on the messages and the inclusion of pre-message measures, as this survey was kept intentionally as brief as possible. However, value was added using measures of mediators.

### Mechanisms for Change in Behavioral Intentions

The second objective of this study involved considering handwashing attitudes and emotions as statistical mediators of the relationship between message reception and handwashing intentions. Thus, the mechanisms by which messaging impacted behavioral intentions were explored. Handwashing emotions, but not attitudes, were found to be a significant mediator of the relationship. This finding is consistent with research comparing affective and cognitive pathways for health behavior, in that the mediation analysis suggests that effect of the exchange message on intentions was due to increased positive emotions around handwashing (26). The mediation by emotions, and not attitudes, highlights that emotions are strong motivators for behavior during health crises such as the current pandemic.

Lastly, as one of the objectives for this study, age was included in the mediational model as a possible moderator. Results suggest that while the exchange message increased handwashing emotions, which then increased handwashing intentions, this only occurred for younger and middle-aged participants. Older adults were not significantly influenced by this message to emotions pathway, perhaps because older participants already reported higher intentions to wash their hands. This finding suggests that social exchange messages for increasing handwashing will be most effective for individuals, such as younger and middle age individuals, with lower initial intentions to wash their hands regularly.

### Theoretical, Social, and Policy Implications

The present study may provide insights about handwashing intentions that could be used in various settings in the future; such insights could inform handwashing messages to address the seasonal flu, influence message types in businesses or restaurants. Future studies could also help inform best-practice approaches for large-scale handwashing interventions in the event of another pandemic. The results of the present study also suggest that a theory-based, social marketing approach could be utilized for a successful health messaging campaign.

### Limitations

The authors must acknowledge potential limitations as well as the strengths described above. Participants were recruited through a third-party recruitment service and were not specifically selected to match the demographic breakdown of the population of the United States. As such, the generalizability to different racial/ethnic populations is reduced. Future studies should examine the effectiveness of these social marketing frames in diverse samples. Additionally, given the atmosphere surrounding COVID-19 in the United States when the questionnaire was administered, participants may have been more receptive to the messages because of saturation of handwashing messaging in all media. The study also utilized self-report measures of behavioral intentions and did not determine how message type translates into actual handwashing behavior. The authors acknowledge limitations exist due to the few constructs used to measure important outcomes; the instrument only contains two items for attitudes, and one item for the stages of change. Due to the rapidity needed to create the questionnaire at the beginning of the pandemic, the authors also acknowledge it was not pre-tested. The present study may be influenced by the specific recruitment procedures involved in data collection, such as use of an online survey instrument, incentives, and self-reported measures. However, this approach was beneficial for obtaining timely data from a national sample on this critical issue during a pandemic, when face-to-face research was restricted. The authors also acknowledge that the messages shown to participants in the online questionnaire are only a few possible framings. It is unknown how different framings might compare to the messages shown in this study. Further exploration is warranted to understand how message framing like loss framing and fear appeals may be viewed by individuals during a pandemic. Additional studies may also benefit from increased sample sizes to improve generalizability.

## Conclusions

These findings have implications for health messaging during public health emergencies. These results suggest that even simple, brief, and easily conveyable messages are able to positively impact behavioral intentions around handwashing during the early stages of a health crisis. Simple exchange messages may be particularly useful in increasing handwashing intentions for individuals with previously low intentions because of their ability to increase emotions toward the behavior. This also suggests that these types of messages may be successful in delivering social marketing messages about handwashing when there is already high awareness in the United States, as they do not rely on changing attitudes or other cognitive variables. Further, based on the mediational analyses, it appears that health messages designed to increase handwashing would benefit by directly targeting emotions.

This study has several strengths that make it an important addition to the scientific community. The authors are unaware of any scientific literature that specifically examine social marketing frames and handwashing messages in the context of COVID-19. This is also the only study the authors are aware of that examines handwashing messages using the Transtheoretical Model during the context of a pandemic. Finally, these data also add to the literature by showing that emotions, but not attitudes, statistically mediate the effect of social exchange messages on handwashing intentions.

## Data Availability Statement

The datasets presented in this study can be found in online repositories. The names of the repository/repositories and accession number(s) can be found below: https://osf.io/835p7/?view_only=6370596c6787415db68e27a4e81816ab.

## Ethics Statement

The studies involving human participants were reviewed and approved by University of Toledo Institutional Review Board. The patients/participants provided their written informed consent to participate in this study.

## Author Contributions

JM was involved in the original design concept of the study, development of handwashing messages, data cleaning, and questionnaire design. KC was involved in the original design and formal data analysis of the study, and developed tables for the manuscript. KF was involved in the original design concept, questionnaire design, and supervision of the study. AG was involved in original design, study supervision, and data analysis. All authors participated in full review and editing of the final manuscript.

## Conflict of Interest

The authors declare that the research was conducted in the absence of any commercial or financial relationships that could be construed as a potential conflict of interest.
