# Supplementary material for: Handwashing Message Type Predicts Behavioral Intentions in the United States at the Beginning of the Global COVID-19 Pandemic
Source: Front Public Health. 2021 May 5;9:583491. doi: 10.3389/fpubh.2021.583491 (PMC8131518; doi:10.3389/fpubh.2021.583491)
Supplement: Supplementary file 2 [file Data_Sheet_2.docx]

**Supplemental Material**

**Measures:**

***Handwashing Attitudes***

**In regards to COVID-19, the novel coronavirus,** please choose the response which best represents how you feel about each statement.

|  | **Strongly disagree** | **Disagree** | **Slightly disagree** | **Neither agree nor disagree** | **Slightly agree** | **Agree** | **Strongly agree** |
| --- | --- | --- | --- | --- | --- | --- | --- |
| Hand washing is effective in preventing disease. |  |  |  |  |  |  |  |
| Hand washing is important. |  |  |  |  |  |  |  |

***Handwashing Emotions/Affect***

**In regards to COVID-19, the novel coronavirus,** please choose the response which best represents how you feel about each statement.

|  | **Strongly disagree** | **Disagree** | **Slightly disagree** | **Neither agree nor disagree** | **Slightly agree** | **Agree** | **Strongly agree** |
| --- | --- | --- | --- | --- | --- | --- | --- |
| I am angry when others do not wash their hands. |  |  |  |  |  |  |  |
| I am proud of washing my hands. |  |  |  |  |  |  |  |
| I would feel guilty if I did not wash my hands. |  |  |  |  |  |  |  |
| I am annoyed when others do not wash their hands. |  |  |  |  |  |  |  |
| Washing my hands gives me control over my health. |  |  |  |  |  |  |  |

***Handwashing Intentions***

**In light of COVID-19, the novel coronavirus,** do you in intend to wash your hands...

|  | **Never** | **Seldom** | **About half of the time** | **Usually** | **Always** |
| --- | --- | --- | --- | --- | --- |
| ...after blowing your nose, coughing or sneezing |  |  |  |  |  |
| ...after visiting a public space |  |  |  |  |  |
| ...after touching surfaces outside of the home, including money |  |  |  |  |  |
| ...before, during and after caring for a sick person |  |  |  |  |  |
| ...before and after eating |  |  |  |  |  |
| ...for at least 20 seconds each time |  |  |  |  |  |

***Handwashing Readiness to Change***

**In response to the novel coronavirus, COVID-19,** please select the most accurate option for your intention to engage in the following behaviors in order to prevent disease and stay healthy.

- I do not intend to do this
- I have thought about doing this, but do not yet plan to.
- I intend to do this but have not yet done it.
- I am actively doing this now.
- This is something I have already done for a long time, and will continue doing, in order to prevent disease.

**Exploratory Analyses**

While hypotheses about variables presented in the present study were made a priori, additional post hoc analyses considering additional demographic moderators to the conditional process relationship are included below.

The mediation of handwashing emotions between message condition and behavioral intentions was not moderated by other demographic variables:

Table: Interaction coefficients, p-values, and indices of moderated-mediation for demographic variables presenting 95% CIs.

|  | Interaction Values | | Indices of Moderated-Mediation | |
| --- | --- | --- | --- | --- |
|  | *b* | *p* | LCI | UCI |
| Income | .001 | .892 | –.001 | .001 |
| Race* | –.040 | .462 | ­–.004 | .009 |
| Health condition increasing vulnerability to COVID-19 | .039 | .506 | –.006 | .007 |

*Note:* **Race was dichotomized to White participants and participants identifying as another race in order to create groups of comparable sizes.*
